# Teledermatology to Facilitate Patient Care Transitions From Inpatient to Outpatient Dermatology: Mixed Methods Evaluation

**DOI:** 10.2196/38792

**Published:** 2022-08-03

**Authors:** Samantha M R Kling, Erika A Saliba-Gustafsson, Marcy Winget, Maria A Aleshin, Donn W Garvert, Alexis Amano, Cati G Brown-Johnson, Bernice Y Kwong, Ana Calugar, Ghida El-Banna, Jonathan G Shaw, Steven M Asch, Justin M Ko

**Affiliations:** 1 Division of Primary Care and Population Health Department of Medicine Stanford University School of Medicine Palo Alto, CA United States; 2 Department of Dermatology Stanford University School of Medicine Stanford, CA United States; 3 Center for Innovation to Implementation (Ci2i) Veterans Affairs Palo Alto Heath Care System Menlo Park, CA United States

**Keywords:** teledermatology, telemedicine, telehealth, video visits, care transitions, care coordination, discharge planning, follow-up, inpatient, outpatient, mixed methods, dermatology, mobile phone, smartphone

## Abstract

**Background:**

Both clinicians and patients have increasingly turned to telemedicine to improve care access, even in physical examination–dependent specialties such as dermatology. However, little is known about whether teledermatology supports effective and timely transitions from inpatient to outpatient care, which is a common care coordination gap.

**Objective:**

Using mixed methods, this study sought to retrospectively evaluate how teledermatology affected clinic capacity, scheduling efficiency, and timeliness of follow-up care for patients transitioning from inpatient to outpatient dermatology care.

**Methods:**

Patient-level encounter scheduling data were used to compare the number and proportion of patients who were scheduled and received in-clinic or video dermatology follow-ups within 14 and 90 days after discharge across 3 phases: June to September 2019 (before teledermatology), June to September 2020 (early teledermatology), and February to May 2021 (sustained teledermatology). The time from discharge to scheduling and completion of patient follow-up visits for each care modality was also compared. Dermatology clinicians and schedulers were also interviewed between April and May 2021 to assess their perceptions of teledermatology for postdischarge patients.

**Results:**

More patients completed follow-up within 90 days after discharge during early (n=101) and sustained (n=100) teledermatology use than at baseline (n=74). Thus, the clinic’s capacity to provide follow-up to patients transitioning from inpatient increased from baseline by 36% in the early (101 from 74) and sustained (100 from 74) teledermatology periods. During early teledermatology use, 61.4% (62/101) of the follow-ups were conducted via video. This decreased significantly to 47% (47/100) in the following year, when COVID-19–related restrictions started to lift (*P*=.04), indicating more targeted but still substantial use. The proportion of patients who were followed up within the recommended 14 days after discharge did not differ significantly between video and in-clinic visits during the early (33/62, 53% vs 15/39, 38%; *P*=.15) or sustained (26/53, 60% vs 28/47, 49%; *P*=.29) teledermatology periods. Interviewees agreed that teledermatology would continue to be offered. Most considered postdischarge follow-up patients to be ideal candidates for teledermatology as they had undergone a recent in-person assessment and might have difficulty attending in-clinic visits because of competing health priorities. Some reported patients needing technological support. Ultimately, most agreed that the choice of follow-up care modality should be the patient’s own.

**Conclusions:**

Teledermatology could be an important tool for maintaining accessible, flexible, and convenient care for recently discharged patients needing follow-up care. Teledermatology increased clinic capacity, even during the pandemic, although the timeliness of care transitions did not improve. Ultimately, the care modality should be determined through communication with patients to incorporate their and their caregivers’ preferences.

## Introduction

In 2020, the COVID-19 pandemic drove telemedicine to the forefront of health care [1,2]; dermatology care was no exception. Prepandemic teledermatology had gained popularity in some specific use cases [3,4]; however, the pandemic gave rise to new policies that overcame previous restrictions to ensure continued access to care, facilitating a rapid pivot to telemedicine for outpatients, including patients transitioning from inpatient care [5-7].

The highly visual nature of dermatology is well suited for this cost-effective and efficient care modality [4,8-10], which is well received by clinicians and patients [10-14]. Although convenience and improved access to care are the primary benefits, especially for rural and underserved populations, teledermatology also boasts time and cost savings, greater flexibility for dermatologists and patients, fewer no-shows, and better continuity of care [4,5,10,15-25]. However, teledermatology has some shortcomings that affect care delivery, including suboptimal image quality, patient privacy, diagnostic accuracy, network connectivity, patient technological literacy, and access to digital devices [8,9,11,15]. In addition, the inaccessibility of in-clinic tools and treatments (eg, dermoscopy, biopsy, and cryotherapy) makes managing certain conditions challenging [9]. These limitations may disproportionally affect patients with low socioeconomic status, Medicare beneficiaries, older adults, and non–English-preferring patients [9,11], who may also be at risk for delayed care transitions.

Nevertheless, teledermatology may be particularly beneficial for patients transitioning from inpatient dermatology consultation services to outpatient dermatology care. Currently, high-risk patients who are hospitalized often experience numerous comorbidities and may experience difficulties accessing in-clinic follow-up care. As a result, they risk receiving fragmented care and being lost to follow-up, which could have serious health consequences [26-28]. Teledermatology may improve follow-up access for these patients by increasing the capacity of dermatology clinics and improving the efficiency of scheduling and care provision. As video visits become a fixture in health care expected by patients and clinicians, it is essential to understand whether teledermatology supports timely care transitions. We retrospectively evaluated teledermatology use and its impact on the clinic’s capacity, scheduling efficiency, and timeliness of follow-up care for patients transitioning from inpatient to outpatient dermatology care and explored dermatology clinicians’ and schedulers’ perceptions of teledermatology for this patient population.

## Methods

### Setting

Stanford University’s Department of Dermatology encompasses 13 outpatient clinics with 16 subspecialties and provides inpatient consultative services in a quaternary hospital; that is, consultation requests placed by the patient’s admitting team, such as general medicine or oncology. Consultations are delivered by 5 dermatologists and 2 dermatology residents on monthly rotations. The team consults >1500 inpatients per year, many of whom have complex, high-risk skin conditions in immunocompromised states and have multiple clinical teams involved in their care. Approximately 40% of these patients require postdischarge outpatient follow-up.

### Intervention: Teledermatology

The department rapidly implemented teledermatology across all ambulatory clinics in response to the statewide COVID-19 stay-at-home orders in March 2020 [10]. Clinicians were provided with video visit–enabled hardware to enable the remote provision of teledermatology. All clinicians and staff completed the web-based training developed for the institution’s rollout. Initially, clinicians and staff were encouraged to convert all nonurgent or emergent in-clinic visits into video visits. Once in-clinic capacity began to expand in spring 2020, department-developed clinical criteria guided appropriate video visit use for all patients except (1) patients with high skin cancer risk requiring full skin examination, including melanoma; (2) patients requiring specialized examinations (scalp and genitals); and (3) patients requiring procedural interventions. As of July 2022, teledermatology had remained a fixture and was offered to patients transitioning from inpatient to outpatient care.

### Mixed Methods

#### Overview

Outcomes derived from quantitative scheduling data and qualitative interviews are defined in Table 1. Data were consolidated throughout the analysis and interpreted in parallel to understand converging and diverging issues regarding teledermatology use and its impact on the clinic's capacity, clinical appropriateness, sustainability, and the remaining barriers for patients transitioning from inpatient to outpatient care.

**Table 1:** Outcomes, definitions, and data sources used to evaluate the use, impact, and sustainability of teledermatology for patients transitioning from inpatient to outpatient dermatology follow-up care.

Outcomes and definitions					Data sources
**Clinic’s capacity**					
	**Number and proportion of patients after discharge**				
		Scheduled follow-up within 90 days after discharge	Patient-level scheduling data		
		Completed follow-up within 90 days after discharge	Patient-level scheduling data		
**Teledermatology use**					
	Number and proportion of follow-up visits completed over video within 90 days after discharge			Patient-level scheduling data	
	Acceptability of teledermatology for postdischarge follow-up patients among clinicians, residents, schedulers, and patients			Clinician and scheduler interviews	
**Clinical appropriateness**					
	Perceived fit or compatibility of teledermatology within this setting, particularly for patients transitioning from inpatient to outpatient dermatology care			Clinician and scheduler interviews	
**Teledermatology to support timely care transitions**					
	**Scheduling efficiency**				
		Days from hospital discharge to initial scheduling for in-clinic and video visits	Patient-level scheduling data		
		Days from hospital discharge to finalized scheduling for in-clinic and video visits	Patient-level scheduling data		
		Perceived impact of teledermatology on scheduling efficiency	Clinician and scheduler interviews		
	**Timeliness of follow-up visits**				
		Days from hospital discharge to follow-up visit completion for in-clinic and video visits	Patient-level scheduling data		
		Number and proportion of patients who attended follow-up within 14 days after discharge (local benchmark)	Patient-level scheduling data		
	**Incomplete follow-up visits**				
		Number and proportion of patients who scheduled but did not complete a teledermatology or in-clinic visit	Patient-level scheduling data		
		Perceived impact of teledermatology on follow-up visit completion	Clinician and scheduler interviews		
**Remaining barriers to video visit coordination**					
	Perceived long-term sustainability of video visits and the barriers need to be addressed to improve clinician, scheduler, and patient experience			Clinician and scheduler interviews	

#### Quantitative: Inclusion Criteria, Data Collection, and Analysis

Patients who received a dermatology consultation in the inpatient or emergency department settings were discharged in 1 of the 3 study periods and potentially needed follow-up with outpatient dermatology. The three study periods were (1) June 1 to September 30, 2019 (baseline [before teledermatology]); (2) June 1 to September 30, 2020 (early teledermatology); and (3) February 1 to May 31, 2021 (sustained teledermatology). Follow-up scheduling and care were recorded for 90 days after discharge; visits scheduled >90 days after discharge were likely unrelated to the patient’s hospitalization. Eligible patients and relevant events were retrospectively identified using the electronic health records and scheduling data. Inpatient dermatology consults were identified using Current Procedural Terminology codes (Multimedia Appendix 1).

Teledermatology use and its impact on the clinic’s capacity, scheduling efficiency, and timeliness of follow-up care were compared across periods and visit modalities (video and in-clinic) using the outcomes described in Table 1. Descriptive statistics were calculated to describe patient characteristics and assess differences across the 3 study periods and by visit modality. Statistical significance was assessed using the Kruskal-Wallis test for patient age, chi-square test for categorical (ie, proportional) outcomes, and generalized linear models for continuous outcomes (eg, days from discharge). Differences in teledermatology use by patient age and distance between patient residence and outpatient dermatology clinic were determined using chi-square tests. Clinically meaningful (<70 years vs ≥70 years) or median-based (<21 miles vs ≥21 miles) categories were used. *P* values were adjusted for multiple comparisons with an adaptive, 2-stage linear step-up procedure, and significance was set at *P*<.05 [29].

#### Qualitative: Data Collection and Analysis

We designed a semistructured interview guide to capture perceptions of teledermatology for follow-up care of patients transitioning from the inpatient setting. Clinicians and schedulers were eligible if they were involved in transitioning patients from inpatient to outpatient dermatology. All eligible clinicians and schedulers (ie, 5 dermatologists, 5 dermatology residents, and 13 schedulers) were invited via email (plus 2 reminders) to participate in a 30-minute phone interview. Ultimately, 15 interviews (5/5, 100% dermatologists; 5/5, 100% residents; and 6/13, 46% schedulers) were conducted between April and May 2021 by 2 experienced qualitative researchers (EAS-G and AA), ranging from 30 to 60 minutes. The interviews were audio recorded and subsequently transcribed.

Data were analyzed deductively and inductively using Microsoft Excel. Deductive codes were derived from the Proctor implementation outcomes [30]. We used multiphase matrix analysis by leveraging rapid analytic procedures to achieve consensus coding of transcripts and extract early themes [31]. EAS-G and AA independently summarized transcripts after each interview; summaries were reviewed, and consensus discussions were held. Summaries were then consolidated into a matrix to identify and compare themes across interviewees. To ensure anonymity, all identifiable information was removed from transcripts, summaries, and reports.

### Ethics Approval

This retrospective quality improvement evaluation received a nonresearch determination by Stanford University’s Institutional Review Board (IRB-60382). Interviewees provided informed verbal consent before initiating the interviews and were assured that all responses would remain confidential. Detailed interview notes were taken if consent for recording was not provided.

## Results

### Patient Characteristics

Patient characteristics are summarized in Table 2. Briefly, 194 patients, 218 patients, and 256 patients were discharged following an inpatient dermatology consultation during the baseline, early teledermatology, and sustained teledermatology phases, respectively. The median patient age was similar across the 3 periods (61.0, 60.5, and 55.5 years for baseline, early teledermatology, and sustained teledermatology, respectively; *P*=.11). Approximately half of the patients lived ≥21 miles from the dermatology clinic during each study phase, and most had public insurance.

**Table 2:** Characteristics of patients who potentially needed outpatient postdischarge follow-up dermatology care following an inpatient dermatology consultation during one of three periods: baseline (N=194), early teledermatology (N=218), and sustained teledermatology (N=256).

Patient characteristics		Baseline (before teledermatology; June to September 2019), n (%)	Early teledermatology (June to September 2020), n (%)	Sustained teledermatology (February to May 2021), n (%)
Patients with inpatient dermatology consultation		194 (100)	218 (100)	256 (100)
**Sex**				
	Female	100 (51.5)	116 (53.2)	135 (52.7)
	Male	94 (48.5)	102 (46.8)	121 (47.3)
**Age group (years)**				
	0-29	19 (9.8)	27 (12.4)	34 (13.3)
	30-49	37 (19.1)	48 (22)	69 (27)
	50-69	83 (42.8)	82 (37.6)	92 (35.9)
	≥70	55 (28.4)	61 (28)	61 (23.8)
**Distance from outpatient clinic (miles)^a^**				
	0-20	90 (46.4)	104 (47.7)	133 (52)
	≥21	104 (53.6)	114 (52.3)	123 (48)
**Insurance type**				
	Private	42 (21.6)	28 (12.8)	40 (15.6)
	Public	148 (76.3)	187 (85.8)	210 (82)
	Other or no insurance identified	4 (2.1)	3 (1.4)	6 (2.3)
**Patient hospital stay**				
	Emergency department	25 (12.9)	40 (18.3)	56 (21.9)
	Inpatient	169 (87.1)	178 (81.7)	200 (78.1)
**Dermatology specialty for follow-up**				
	Dermatology	63 (32.5)	91 (41.7)	97 (37.9)
	Dermato-oncology	39 (20.1)	41 (18.8)	31 (12.1)
	No follow-up	92 (47.4)	86 (39.4)	128 (50)

^a^Median distance between the patient’s zip code and the primary dermatology clinic in Palo Alto, CA, United States, was 21 miles.

### Clinic Capacity

**Figure 1:**
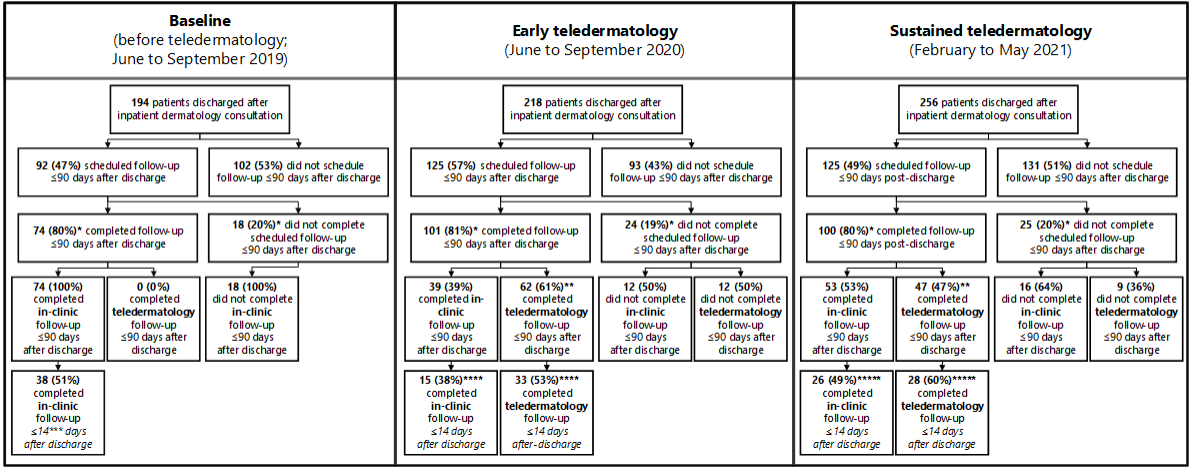
Number and percentage of patients discharged following an inpatient dermatology consultation and who were scheduled for and completed an outpatient dermatology follow-up visit within 90 days after discharge in the clinic or via video. *P=.99 indicating no difference between baseline, early teledermatology, and sustained teledermatology phases. **P=.04 indicating significant difference between the early and sustained teledermatology phases. ***A local benchmark. ****P=.15 indicating no difference between the teledermatology and in-clinic follow-ups during the early teledermatology phase. *****P=.29 indicating no difference between the teledermatology and in-clinic follow-ups during the sustained teledermatology phase.

More patients were scheduled for outpatient dermatology visits within 90 days after discharge during the early (n=125) and sustained (n=125) teledermatology phases than at baseline (n=92), indicating a 36% increase (n=125 from 92 and n=125 from 92 patients for early and sustained teledermatology, respectively) in the scheduling capacity (Figure 1). Similarly, the number of follow-up visits completed within 90 days after discharge was higher in the early teledermatology (n=101) and sustained teledermatology (n=100) than at baseline (n=74), indicating a sustained increase in capacity. The proportion of patients who completed their follow-up did not differ across the 3 evaluation periods, as shown in Figure 1.

### Teledermatology Use

Teledermatology use was highest in the early teledermatology period, with 61.4% (62/101) of follow-ups completed via video. This decreased significantly to 47% (47/100) of follow-ups in the sustainability period (*P*=.04), indicating a more targeted yet still substantial use (Figure 1). Interviewees remarked that patient acceptance of teledermatology and technology capabilities varied during early implementation; however, acceptance increased as it became the standard of care (see exemplary quotes in Textbox 1).

Clinicians and schedulers believed that older patients may prefer clinic visits, whereas patients residing farther from the clinic may favor video visits (Textbox 1). However, video visit use did not differ by patient age during the early or sustained teledermatology phase or by distance from the clinic during the early teledermatology phase (Table 3). However, in the sustained period, significantly more patients residing ≥21 miles away from the clinic had follow-up video visits (26/41, 63% patients) than those living closer (21/59, 36% patients; *P*=.01), confirming the interviewees’ view that patients living farther away preferred video to in-clinic visits as teledermatology became optional (ie, no longer mandated because of the pandemic).

**Exemplary quotes from interviews with dermatologists, residents, and scheduling staff describing the use of teledermatology for patients transitioning from inpatient to outpatient dermatology care.**

**Teledermatology use**

- “In the pandemic, people were frequently upset that they had to do a video visit. They wanted to be seen in person [...] Now that people are more used to the virtual world, they seem to be more okay with doing video visits. But I still think that for certain people, they just really don’t want anything to do with them [...]” [Resident 5]
- “...[video visits] made things a little bit easier because we are able to get over that hurdle of travel. So for a patient that maybe is hours away where they’re just never going to come back for a 15-30 minute, dermatology visit, where they’re just not able to do that. It has made us better able to at least connect with them.” [Dermatologist 3]

**Clinical appropriateness**

- “...for some patients video visits are totally fine, like a patient comes in with a drug rash, they’re totally better, you’re just checking it and making sure they’re not flaring again. It’s perfect. But when there’s an issue where they might need a culture or a lab, or they need a little more intensive care, like wound change or something like that, it’s very difficult. It’s challenging. Sometimes we just need in-person.” [Dermatologist 3]
- “...for some of our sick patients, maybe their skin isn’t their priority, and it isn’t a very complex thing that requires inpatient evaluation; it saves them so much time and stress, and also, kind of helps close the loop on our end as well.” [Dermatologist 2]
- “...medically, from our perspective, people that are in hospital tend to have certain conditions so a close follow-up where we can actually see all their skin, as opposed to pictures, is more helpful.” [Resident 4]
- “...a decent amount of the time we’ll do video visits, but we are leaving it up to patients. So even if it’s something that’s not really serious, they’d rather be seen in person, we’ll still accommodate them in person.” [Resident 5]

**Teledermatology to support timely care transitions**

- “A lot of the clinicians have more video visits than they do in person so it’s a lot easier just to get them in the video.” [Front office scheduler 2]
- “I think it [video visits] makes our work a little easier because we have more options to give the patient...[video visits] give the patient more options because sometimes patients don’t want to come into clinic, they’d rather do a video. [...] on top of that, with video visits, we can get the patients in sooner because with a lot of the video visits, there’s more video visits available where we can get the patients in sooner versus in-person that are booking months out.” [New patient coordinator 3]
- “The biggest difference for me is provider availability particularly for patients that live pretty far away because they’re able to be more flexible in terms of when they can schedule and then they can schedule sooner.” [Resident 3]
- “...it’s probably easier to schedule video visits. I think attendings can squeeze them in a bit faster than in-person. [...] having the option makes it a little easier to schedule in a timely manner. That’s really the only benefit I can think of.” [Resident 5]
- “...no-show rate is much higher for video visits ... significantly higher.” [Resident 5]
- “I find the video visits are a lot easier because they’re more likely to follow-up, because a lot of patients otherwise don't show when they have to come in-person.” [Dermatologist 5]

**Remaining barriers to video visit coordination**

- “...ideally, if they could leave the hospital with a follow-up appointment, that would actually help even more, but most of the time, that’s just not feasible.” [Resident 3]
- “I...prefer in-person visits to video visits just because you’re relying a lot on the patient uploading photos and then the photos they upload have to be good quality... I have definitely been fooled before where I see photos that a patient’s taken and thought one thing and then when you see them in person, it’s much different. So I think there definitely are limitations to video visit.” [Resident 5]
- “...we have a very large elderly group of patients and elderly people aren’t tech savvy. They’re a main group of people who are vulnerable during COVID but it made it really hard for them to do video visits.” [Front office scheduler 1]
- “...there are barriers, like if we’re not getting the best photos from the patient, or let’s say they have to do labs. There’s a lot of really tricky coordination. They need a lab slip and we have to get it to them somehow. Then they have to get to the lab and call us since we can’t call the lab for the results, and we’re really reliant on their primary care doctors, if they have one.” [Clinician 3]

**Table 3:** Completion of in-clinic and video outpatient dermatology postdischarge follow-up visits (by patient age and location) for patients who received an inpatient dermatology consultation and were discharged across 3 periods: baseline, early teledermatology, and sustained teledermatology.

Follow-up visit modality		Baseline (before teledermatology; June to September 2019)		Early teledermatology (June to September 2020)				Sustained teledermatology (February to May 2021)			
		Total visits, N	In-clinic, n (%)	Total visits, N	In-clinic, n (%)	Video, n (%)	*P* value^a^	Total visits, N	In-clinic, n (%)	Video, n (%)	*P* value
**Patient age (years)**							.32				.47
	<70	56	56 (100)	72	30 (42)	42 (58)		71	36 (51)	35 (49)	
	≥70	18	18 (100)	29	9 (31)	20 (69)		29	17 (59)	12 (41)	
**Distance from clinic (miles)^b^**							.15				.01
	<21	36	36 (100)	47	19 (40)	28 (60)		59	38 (64)	21 (36)	
	≥21	38	38 (100)	54	20 (37)	34 (63)		41	15 (37)	26 (63)	

^a^Differences in proportion between in-clinic and video visits were determined using chi-square tests.

^b^The median distance between the patient’s zip code and the primary dermatology clinic in Palo Alto, CA, United States was 21 miles.

### Clinical Appropriateness

Clinicians expressed interest in continuing to offer video visits to recently discharged patients who have ongoing stable conditions that are well-suited to teledermatologic care or limitations preventing in-clinic care (Textbox 1). Teledermatology was perceived to reduce barriers (eg, lack of time or resources to travel) to attending follow-up in person for high-risk patient populations who may need to attend several follow-up visits and may consequently deprioritize their dermatological issues. A video visit was perceived as better than no follow-up at all for patients who were too debilitated to travel, even if an in-clinic visit was clinically ideal.

Video visits were often considered ideal for quick, simple check-ins for postdischarge patients as they had recently been examined in person, whereas in-clinic visits were considered more appropriate for conditions requiring skin examinations or procedures, laboratory tests, or dressing changes. Nevertheless, a few residents believed that video visits were less suitable for postdischarge patients (Textbox 1). However, most agreed that the choice should ultimately be the patient’s own. According to clinicians, patients appreciate having a choice that meets their priorities, needs, and preferences, and accommodating patients to complete the necessary follow-up is worthwhile.

### Teledermatology to Support Timely Care Transitions

To understand the impact of teledermatology on the scheduling efficiency and timeliness of care transitions, differences in days from discharge to initial scheduling, finalized scheduling, and completion of an outpatient dermatology follow-up visit across each study period and visit modality were assessed (Table 1). The proportion of incomplete scheduled visits is also reported by the study period and visit modality.

#### Scheduling Efficiency

The average days from hospital discharge to initial scheduling of in-clinic visits increased from 4.2 (SD 7.2) days at baseline to 8.5 (SD 10.2) days during early teledermatology (*P*=.01; Table 4). This returned to 3.0 (SD 4.8) days in the sustained period, similar to baseline (*P*=.40). In contrast, the average number of days for initial scheduling of video visits was 4.3 (SD 6.8) days and 3.7 (SD 10.1) days for the early and sustainability periods, respectively, similar to the 4.2 (SD 7.2) days at baseline when only in-clinic visits were offered (*P*=.89 and *P*=.76, respectively; Table 4). The results were similar for the other efficiency measures; that is, days from discharge to final scheduling (Table 4). These results potentially reflect the decreased availability and increased difficulty in scheduling in-clinic visits in the early implementation period due to pandemic-related restrictions and concerns; however, the increased flexibility and appointment availability of teledermatology enabled comparable timeliness of follow-up scheduling during the pandemic as to before the pandemic, as reported by schedulers (Textbox 1).

**Table 4:** Days from inpatient discharge to initial scheduling, final scheduling, and completion of outpatient dermatological follow-up visits for patients who received an inpatient dermatology consultation and were discharged during 3 periods: baseline, early teledermatology, and sustained teledermatology.

Follow-up visit modality		Baseline (before teledermatology; June to September 2019)	Early teledermatology (June to September 2020)				Sustained teledermatology (February to May 2021)			
		In-clinic	In-clinic	*P* value^a^	Video	*P* value	In-clinic	*P* value	Video	*P* value
Patients who completed follow-up ≤90 days after discharge, N		74	39	N/A^b^	62	N/A	53	N/A	47	N/A
**Days from inpatient discharge, mean (SD)**										
	Initial scheduling of outpatient follow-up	4.2 (7.2)	8.5 (10.2)	.01	4.3 (6.8)	.89	3.0 (4.8)	.40	3.7 (10.1)	.76
	Final scheduling of outpatient follow-up	9.0 (13.5)	14.9 (17.8)	.02	6.4 (7.6)	.22	7.6 (11.2)	.51	5.7 (12.3)	.18
	Completed outpatient follow-up	19.0 (14.8)	25.5 (21.9)	.19	17.3 (15.1)	.94	19.0 (17.7)	.99	18.4 (19.4)	.94

^a^Difference from baseline was determined using least squares means in generalized linear regression and was adjusted for multiple comparisons.

^b^N/A: not applicable.

#### Timeliness of Follow-up Visits

The average number of days from hospital discharge to completed follow-up visits did not differ across the periods or by visit modality (Table 4). A higher, although nonsignificant, proportion of patients was seen within 14 days after discharge, which is a local follow-up benchmark, via video than in the clinic (Figure 1). During the early teledermatology period, 53% (33/62) of follow-ups were conducted using teledermatology, whereas 38% (15/39) of follow-ups were conducted in the clinic (*P*=.15). Similarly, 60% (28/47) of follow-ups were conducted using teledermatology, whereas 49% (26/53) of follow-ups were conducted in the clinic (*P*=.29) during the sustained teledermatology period (Figure 1). Thus, although teledermatology follow-up visits were scheduled slightly faster (although not significant) than prepandemic in-clinic visits, this did not result in timelier follow-up care.

#### Incomplete Follow-up Visits

Relatively few patients missed their scheduled follow-up visits during the 3 periods (Figure 1). Of the 24 patients who missed their scheduled visit in the early teledermatology period, 12 (50% patients) had a scheduled teledermatology visit and 12 (50%) had scheduled in-clinic visits. A total of 25 patients did not complete their scheduled visit during the sustained teledermatology period, of whom 9 (36% patients) were scheduled for teledermatology and 16 (64% patients) for an in-clinic visit. This aligned with clinician and resident perceptions that teledermatology facilitated the completion of follow-up care but may not improve cancelation rates (Textbox 1). Some perceived that cancelation rates were higher for video visits, and others said they were higher for clinic visits; the small number of missed visits limits our evaluation of these perceptions.

### Remaining Barriers to Video Visit Coordination

Interviewees acknowledged the benefits of teledermatology but indicated that care coordination and video visit setup were sometimes challenging (Textbox 1). Access to a smartphone and a means of taking a high-quality photograph were considered essential, especially for at-risk populations, including older adults, who were believed to benefit the most from improved access through reduced travel and risk during the pandemic. Schedulers perceived that these patients frequently needed help in setting up their devices and uploading their photographs before a visit. Despite this assistance, the photographs submitted sometimes lacked sufficient quality. Care coordination via video was further complicated if the patients required laboratory tests. Some interviewees suggested that this coordination should begin during hospitalization at the patient’s bedside to integrate and prioritize the care needs of patients and caregivers in discharge planning and follow-up care scheduling.

## Discussion

### Principal Findings

Teledermatology was frequently used during the evaluation period; two-thirds of the visits were conducted via teledermatology early in the pandemic, whereas about half of the visits continued to be conducted using teledermatology later in the pandemic, indicating more targeted but nevertheless substantial use. Teledermatology availability increased the clinic’s follow-up scheduling capacity for patients transitioning from inpatient to outpatient dermatology care. Teledermatology also provided a flexible option that increased overall clinic capacity while retaining comparable scheduling efficiency and timeliness of care as before the pandemic, even amid a pandemic and strained health care system. However, the scheduling efficiency and timeliness of care transitions did not improve, suggesting that a wider range of efforts are needed to improve these issues. Interviewees viewed teledermatology as an important care modality for providing accessible care, especially for patients with competing medical priorities and limited ability or availability to travel to the clinic, although important logistical and technological limitations were acknowledged for some patients. Ultimately, interviewees believed that patients should make the final choice between in-clinic or video visits.

### Comparison With Prior Work

Teledermatology is an important tool for building clinic capacity, as well as improving scheduling timeliness and completion of care [17,18]. A study in an urban safety net hospital setting found that teledermatology implementation increased the total number of cases evaluated per month by approximately 20% and decreased the time to consultation for new patients from 84.6 days to 6.7 days before the COVID-19 pandemic [17]. Teledermatology has also been shown to increase access to and expedite care for patients in many settings, including referrals from primary to specialty care [19,20], within the Veterans Affairs system [21], in medically underserved populations [22,23], and for those needing inpatient consultations [24]. Our study builds on this literature by demonstrating a sustained increased clinic capacity of 36% after implementing teledermatology in the context of recently discharged patients needing follow-up care; the increase in clinic capacity did not come at the cost of less timely care. In addition, teledermatology services allowed safe access to care during the height of the pandemic when in-clinic care was delayed.

Nevertheless, our study was unable to detect improvements in the timeliness of care, which may be because of the urgency of our patient referrals (desired timeline from discharge to follow-up of only 14 days), as has been reported elsewhere [17-24]. Previous studies that found that video visit implementation improved care timeliness have been conducted in settings where patient referrals were nonurgent [17-24]. Although teledermatology supports increased access to outpatient care for patients of dermatology in general and those needing follow-up care after hospitalization, further research is needed to determine whether telemedicine itself supports more timely scheduling and care provision, particularly for care transitions.

Telemedicine has also been shown to promote visit completion and reduce patient cancelations and no-shows compared with in-clinic visits [16,25]. In the outpatient setting of a large academic health care system, 20% of telemedicine visits were canceled compared with 31% of in-clinic visits [16]. Similarly, a study focusing on dermatological care also showed that a lower percentage of virtual consults, specifically e-consults, were either canceled or not attended (ie, no show) than ambulatory consults (18% vs 39%) [25]. In this study, a few patients missed their scheduled follow-up, of whom 50% (12/24) missed a video visit in the early teledermatology period and 36% (9/25) missed a video visit in the sustained period. This latter result, although a different metric, suggests that recently discharged patients may be less likely to miss scheduled video visits than scheduled in-clinic visits, aligning with previous research [16,25]. However, the small sample size limits interpretability, and additional investigations are needed.

Teledermatology is well suited and highly accepted in dermatology, even for high-risk, recently discharged patients [10-14]. In this study, clinicians and scheduling staff recognized that teledermatology is convenient for patients experiencing difficulties related to their current health, with competing medical and care needs, or limited time and resources to access in-person care. In fact, even as in-clinic visits became more available, patients who lived farther from the clinic were significantly more likely to use teledermatology care than those living closer to the clinic, aligning with previous research reporting on the convenience of telemedicine for rural and underserved populations [10-14]. The widely reported flexibility of teledermatology [4,8-10] was recognized to support care transitions and continuity by interviewed clinicians and schedulers. Ultimately, telemedicine may be particularly well suited to this highly visual specialty, as reported here and elsewhere [4,8-10], perhaps even more so for follow-up care of patients recently physically examined and for whom the clinician expects but wants to confirm an improvement in their condition.

Although video visits are a well-accepted and widely used technology, some patients, clinicians, and schedulers still prefer in-person visits due to their limitations [32-34]. As reported here and previously, teledermatology continues to have shortcomings that affect care delivery, including incomplete previsit preparation, poor quality images, limited patient technological literacy, inability to access certain in-person tools and procedures, and patients’ lack of capabilities with digital devices [8,9,11,15]. Patient privacy concerns, diagnostic accuracy, and network connectivity are also well-recognized limitations [8,9,11,15]. The reported inequities in access to telemedicine care, known as the digital divide [9,11], compound the limitations of teledermatology. Although patients with complex medical issues may particularly benefit from the convenience and flexibility of teledermatology care, they may also lack access to a smartphone and the technological capacity to, for example, take and submit high-quality photographs. Thus, efforts to improve care transitions need to not only be attuned to patients who may benefit from teledermatology but also be able to assess whether such patients have access to the needed skills and technology; if not, alternatives or appropriate support for skills and technology must be provided to facilitate equitable access to care for all. Bedside communication for more patient-centered care [35] or employing dedicated care coordinator teams [28,36,37] to ensure that patients’ care needs are met could not only better support timely care transitions but also ensure high levels of patient and caregiver satisfaction, improved patient outcomes, and lower readmissions.

### Limitations

This retrospective study has 3 main limitations. First, it was conducted in a single health care setting. Second, Current Procedural Terminology codes (Multimedia Appendix 1) associated with inpatient dermatology consultations were used as a surrogate to identify patients potentially needing follow-up care; ideally, such data would be based directly on clinical recommendations, including the follow-up timeline; however, such data were not systematically available. Third, 2 of the 3 study periods were during the COVID-19 pandemic. We considered many factors that could have influenced our findings, including seasonality, COVID-19 pandemic surges and restrictions, and the presence of other quality improvement initiatives, to identify comparable periods. However, this retrospective evaluation was not able to account for all potential confounding factors, including regular policy changes and vaccine availability.

### Conclusions and Future Directions

Telemedicine has moved to the forefront of health care delivery and is anticipated to continue to expand. As telemedicine becomes an established care modality, additional evaluation of its quality, acceptability, and appropriateness for specific use cases and patient populations is needed to ensure the provision and sustainability of appropriate, high-quality care without continuing to widen the care access divide. Teledermatology was viewed as an important tool for maintaining accessible, flexible, and convenient care for patients transitioning from inpatient to outpatient dermatology care. Despite its shortcomings, including photograph quality and varying patient technological capabilities, teledermatology is predicted to be a standard option for patients. However, teledermatology alone does not completely solve care transition delays; it must be coupled with other efforts to improve communication between patients and care teams, patient access to and comfort with video technology, and workflows that support timely and equitable access to follow-up care. Care transitions are a vulnerable time for patients who may easily slip through the cracks and remain a challenge in health care systems [26,28,38-41]. Continued evaluation of alternate approaches to care delivery during care transitions, including telemedicine, as well as reporting of these efforts, is needed to understand their impact on this risky time in the patient care continuum.

## Appendix

Multimedia Appendix 1 Current Procedural Terminology codes associated with inpatient dermatology to identify patients who had an inpatient dermatology consultation and who may have needed outpatient dermatology follow-up care.

## Data Availability

Data that support these findings are available from the corresponding author upon reasonable request.

## References

[1] Bashshur R, Doarn CR, Frenk JM, Kvedar JC, Woolliscroft JO (2020). Telemedicine and the COVID-19 pandemic, lessons for the future. Telemed J E Health.

[2] Myers US, Birks A, Grubaugh AL, Axon RN (2021). Flattening the curve by getting ahead of it: how the VA healthcare system is leveraging telehealth to provide continued access to care for rural veterans. J Rural Health.

[3] Pasquali P, Romero-Aguilera G, Moreno-Ramírez D (2021). Teledermatology before, during, and after the COVID-19 pandemic. Actas Dermosifiliogr (Engl Ed).

[4] Lee JJ, English JC (2018). Teledermatology: a review and update. Am J Clin Dermatol.

[5] Hamad J, Fox A, Kammire MS, Hollis AN, Khairat S (2021). Evaluating the experiences of new and existing teledermatology patients during the COVID-19 pandemic: cross-sectional survey study. JMIR Dermatol.

[6] Perkins S, Cohen JM, Nelson CA, Bunick CG (2020). Teledermatology in the era of COVID-19: experience of an academic department of dermatology. J Am Acad Dermatol.

[7] Asabor EN, Bunick CG, Cohen JM, Perkins SH (2021). Patient and physician perspectives on teledermatology at an academic dermatology department amid the COVID-19 pandemic. J Am Acad Dermatol.

[8] Beer J, Hadeler E, Calume A, Gitlow H, Nouri K (2021). Teledermatology: current indications and considerations for future use. Arch Dermatol Res.

[9] Berman HS, Shi VY, Hsiao JL (2020). Challenges of teledermatology: lessons learned during COVID-19 pandemic. Dermatol Online J.

[10] Pathipati AS, Ko JM (2016). Implementation and evaluation of Stanford Health Care direct-care teledermatology program. SAGE Open Med.

[11] Farr MA, Duvic M, Joshi TP (2021). Teledermatology during COVID-19: an updated review. Am J Clin Dermatol.

[12] Mizes A, Vainder C, Howerter SS, Hu A, Liu A, Harris A, Moorhead A, Falo Jr LD, English 3rd JC (2021). Access to consultative dermatologic care via physician-to-physician asynchronous outpatient teledermatology. Am J Manag Care.

[13] Stadler PC, Senner S, Frey S, Clanner-Engelshofen BM, H Frommherz L, French LE, Reinholz M (2021). Teledermatology in times of COVID-19. J Dermatol.

[14] Mounessa JS, Chapman S, Braunberger T, Qin R, Lipoff JB, Dellavalle RP, Dunnick CA (2018). A systematic review of satisfaction with teledermatology. J Telemed Telecare.

[15] Haque W, Chandy R, Ahmadzada M, Rao B (2021). Teledermatology after COVID-19: key challenges ahead. Dermatol Online J.

[16] Kubes JN, Graetz I, Wiley Z, Franks N, Kulshreshtha A (2021). Associations of telemedicine vs. in-person ambulatory visits and cancellation rates and 30-day follow-up hospitalizations and emergency department visits. Prev Med Rep.

[17] Zakaria A, Maurer T, Su G, Amerson E (2019). Impact of teledermatology on the accessibility and efficiency of dermatology care in an urban safety-net hospital: a pre-post analysis. J Am Acad Dermatol.

[18] Hsiao JL, Oh DH (2008). The impact of store-and-forward teledermatology on skin cancer diagnosis and treatment. J Am Acad Dermatol.

[19] Whited JD, Hall RP, Foy ME, Marbrey LE, Grambow SC, Dudley TK, Datta S, Simel DL, Oddone EZ (2002). Teledermatology's impact on time to intervention among referrals to a dermatology consult service. Telemed J E Health.

[20] Carter ZA, Goldman S, Anderson K, Li X, Hynan LS, Chong BF, Dominguez AR (2017). Creation of an internal teledermatology store-and-forward system in an existing electronic health record: a pilot study in a safety-net public health and hospital system. JAMA Dermatol.

[21] Bezalel S, Fabri P, Park HS (2015). Implementation of store-and-forward teledermatology and its associated effect on patient access in a Veterans Affairs dermatology clinic. JAMA Dermatol.

[22] Naka F, Lu J, Porto A, Villagra J, Wu ZH, Anderson D (2018). Impact of dermatology eConsults on access to care and skin cancer screening in underserved populations: a model for teledermatology services in community health centers. J Am Acad Dermatol.

[23] Leavitt ER, Kessler S, Pun S, Gill T, Escobedo LA, Cockburn M, Sutton A, Crew AB (2016). Teledermatology as a tool to improve access to care for medically underserved populations: a retrospective descriptive study. J Am Acad Dermatol.

[24] Georgesen C, Karim SA, Liu R, Moorhead A, Falo Jr LD, English 3rd JC (2020). Inpatient eDermatology (Teledermatology) can help meet the demand for inpatient skin disease. Telemed J E Health.

[25] Wang RF, Trinidad J, Lawrence J, Pootrakul L, Forrest LA, Goist K, Levine E, Nair S, Rizer M, Thomas A, Wexler R, Kaffenberger BH (2020). Improved patient access and outcomes with the integration of an eConsult program (teledermatology) within a large academic medical center. J Am Acad Dermatol.

[26] Mitchell SE, Laurens V, Weigel GM, Hirschman KB, Scott AM, Nguyen HQ, Howard JM, Laird L, Levine C, Davis TC, Gass B, Shaid E, Li J, Williams MV, Jack BW (2018). Care transitions from patient and caregiver perspectives. Ann Fam Med.

[27] Demiris G, Kneale L (2015). Informatics systems and tools to facilitate patient-centered care coordination. Yearb Med Inform.

[28] Berry LL, Rock BL, Smith Houskamp B, Brueggeman J, Tucker L (2013). Care coordination for patients with complex health profiles in inpatient and outpatient settings. Mayo Clin Proc.

[29] Benjamini Y, Krieger AM, Yekutieli D (2006). Adaptive linear step-up procedures that control the false discovery rate. Biometrika.

[30] Proctor E, Silmere H, Raghavan R, Hovmand P, Aarons G, Bunger A, Griffey R, Hensley M (2011). Outcomes for implementation research: conceptual distinctions, measurement challenges, and research agenda. Adm Policy Ment Health.

[31] Averill JB (2002). Matrix analysis as a complementary analytic strategy in qualitative inquiry. Qual Health Res.

[32] Marchell R, Locatis C, Burgess G, Maisiak R, Liu WL, Ackerman M (2017). Patient and provider satisfaction with teledermatology. Telemed J E Health.

[33] Pearlman RL, Le PB, Brodell RT, Nahar VK (2021). Evaluation of patient attitudes towards the technical experience of synchronous teledermatology in the era of COVID-19. Arch Dermatol Res.

[34] Hadeler E, Gitlow H, Nouri K (2021). Definitions, survey methods, and findings of patient satisfaction studies in teledermatology: a systematic review. Arch Dermatol Res.

[35] (2015). Engaging patients in communication at transitions of care. Australian Commission on Safety and Quality in Health Care (ACSQHC).

[36] Cropley S, Sandrs ED (2013). Care coordination and the essential role of the nurse. Creat Nurs.

[37] Doty MM, Fryer AK, Audet AM (2012). The role of care coordinators in improving care coordination: the patient's perspective. Arch Intern Med.

[38] Miceli A, Krishnamurthy K (2020). Use of a dermatology-specific discharge form to improve outpatient follow-up after inpatient dermatology consultation. J Am Acad Dermatol.

[39] Bumpas J, Copeland DJ (2021). Standardizing multidisciplinary discharge planning rounds to improve patient perceptions of care transitions. J Nurs Adm.

[40] Zakaria A, Chang AY, Kim-Lim P, Arakaki R, Fox LP, Amerson EH (2022). Predictors of postdischarge follow-up attendance among hospitalized dermatology patients: disparities and potential interventions. J Am Acad Dermatol.

[41] Donaho EK, Hall AC, Gass JA, Elayda MA, Lee VV, Paire S, Meyers DE (2015). Protocol-driven allied health post-discharge transition clinic to reduce hospital readmissions in heart failure. J Am Heart Assoc.

