# Supplementary material for: Teledermatology to Facilitate Patient Care Transitions From Inpatient to Outpatient Dermatology: Mixed Methods Evaluation
Source: J Med Internet Res. 2022 Aug 3;24(8):e38792. doi: 10.2196/38792 (PMC9386584; doi:10.2196/38792)
Supplement: Multimedia Appendix 1 [file jmir_v24i8e38792_app1.docx]

## Multimedia Appendix 1. CPT codes associated with inpatient dermatology to identify patients who had an inpatient dermatology consult and who may have needed outpatient dermatology follow-up care.

| **Consult type** | **CPT codes associated with consult type** |
| --- | --- |
| **E-consult^1^** | 99446, 99447, 99448, 99449, 99451 |
| **In-person** | 99251, 99252, 99253, 99254, 99255, 99221, 99222, 99223, 99224, 99225 |

^1^E-consult is a video visit is an asynchronous consultation that is deployed and used in the inpatient setting as a modality to provide dermatology consultative care during hospitalization.
